# Leveraging electronic logistics management information systems to enhance and optimize supply chain response during public health emergencies: lessons from COVID-19 response in Uganda

**DOI:** 10.1186/s40545-023-00517-4

**Published:** 2023-01-17

**Authors:** John Hans Wasswa, Henry Oundo, Martin Olowo Oteba, Henry Komakech, Irene Ochola, Sheila Mwebaze, Denis Okidi, Anthony Kirunda, Shamim Nakadde, Neville Okuna Oteba, Eric Lugada

**Affiliations:** 1 Management Sciences for Health, Uganda, Plot 15, Princess Anne Drive, Bugolobi, P. O. Box 71419, Kampala, Uganda; 2 Ministry of Health, Uganda, Plot 6, Lourdel Road, Wandegeya, P. O. Box 7272, Kampala, Uganda

**Keywords:** Supply chain, COVID-19, Public health emergencies, Pandemics, Electronic logistics management information systems, Uganda

## Abstract

**Background:**

Health supply chain is crucial for proper functioning of a health system and advancing national and international health security goals. The Coronavirus 2019 pandemic caused major challenges for health supply chain systems in Uganda and globally.

**Objectives:**

This study involved literature review to examine how the electronic logistics management information system and related digital systems were harnessed be best support public health emergencies.

**Methods:**

We describe how the health supply chain system leveraged the emergency Electronic Logistic Management Information System developed during the Ebola epidemic in 2019 to support the COVID-19 response in Uganda. The findings are based on the analysis of reports, guidelines, and discussions with stakeholders involved in implementing the electronic Management Information System during the COVID-19 response. Lessons and experiences are shared on how the system supported data visibility, use and health commodity management.

**Results:**

A web-based emergency Electronic Management Information System was developed to support the supply chain system during preparedness and response to the Ebola Virus. The system facilitated coordination, information management and provided real-time data for planning, decision making, and distribution of commodities during the COVID-19 response. To address any human resource challenges, 863 staff were trained and mentored in the use of the system. The system enabled the Ministry of Health to track the distribution of Medical Counter Measures through the warehouses, eight regional pre-positioning centers, and over 2000 user units in 136 district vaccine stores. In addition, the system provided quality data for the quantification and monitoring of commodities at all levels of care. Over 1800 bulk orders were processed through the system. Real time stock status reports were transmitted from all national, regional, district and health facility levels. Procurement tracking reports, stock gap analysis and partner contribution were all accessible and visible in the system. This supported the Ministry of Health’s resource mobilization and coordination efforts.

**Conclusions:**

Availability of reliable, quality real-time data are essential for effective decision making during public health emergencies. The emergency Electronic Logistic Management Information Systems supported health authorities to mount coordinated and effective responses to ensure timely availability of commodities and supplies to support the COVID-19 pandemic response. Lessons learnt from the Ebola epidemic response were translated into actions that enabled effective preparedness and response to the COVID-19 pandemic.

## Background

Emerging infectious diseases (EID) have increased over the past decade [1]. Approximately one human new human infectious disease has emerged every 8 months. Since 1980, more than 35 EIDs have emerged [2]. Uganda is no stranger to infectious disease outbreaks. Over the past two decades, the country has experienced a range of epidemics and re-emerging infectious diseases including Ebola virus Disease (EVD), Crimean-Congo hemorrhagic fever (CCHF), Marburg, anthrax, yellow fever, meningitis, Rift Valley fever, among others. On 11 March 2020, the World Health Organization (WHO) declared COVID-19 a global pandemic [3]. As of November 2022, 635 million confirmed cases had been reported and 6.6 million deaths. As of November 2022, Uganda had reported more than 169,568 confirmed cases and 3630 COVID-19-related deaths [4]. The outbreak resulted in catastrophic effects and astronomical costs associated with managing the epidemic, resulting in ill health, the economic and well-being [5, 6].

Anticipating and preparation for prevention, response for epidemics control are essential epidemic management and control [7]. However, in many Low-and-Middle-Income-Countries (LMICs) multiple factors limit effective planning, preparedness, and response to Public Health Emergencies (PHE) [8, 9]. Other factors including poor housing, inadequate awareness and risk aversion behaviors, poor water, and sanitation services place people at an increased risk of poor health outcomes in the event of epidemic outbreaks [10–12]. The greatest challenge lies in inadequate capacity of the health system to absorb the increasing numbers of severely ill patients [13]. Many LMICs with weak health systems continue to struggle to manage a heavy burden of infectious diseases [14]. In fact, health systems fragility in LMICs has never been of greater importance as has been demonstrated during the COVID-19 pandemic [15]. The COVID-19 pandemic has worsened these challenges leading to inadequate funding, human resource shortages, limited institutional capacity, and weak information systems [16, 17]. These challenges diminish the effectiveness and responsiveness of the health system during the health crisis. Given the sudden increase in demand for services including Essential Medicines and Health Supplies (EMHS) an extra burden is exerted on sub-optimal health system severely disrupting other essential health services [18].

Effective preparedness and response to pandemics require uninterrupted supply of EMHS [19]. However, this is affected by several factors including inadequate funding, weak infrastructure and other support systems [20, 21]. Lack of reliable data to support evidence-based decision-making may lead to short comings in preparedness and response measures [22]. This may lead to poor planning, ineffective and inefficient use of resources. The supply chain system requires routine, timely and accurate data on epidemic profile and commodity availability to support effective and efficient decision making. Availability of data makes it possible for SC stakeholders to assess the risks of supply chain disruptions and take proactive actions. This influences decisions critical to sustaining efficient supply chain for PHEs and ensuring availability of EMHS across the health systems.

We describe the use of an integrated emergency Electronic Logistic Management Information System (eELMIS) to support the supply chain system during the COVID-19 preparedness and response in Uganda. Lessons learnt and experiences are shared on how the eELMIS supported data visibility and use across the supply chain system. The eELMIS includes components of the routine ELMIS and PHE supplies enhancement. The incorporation of PHE epidemic related supplies within the ELMIS facilitated evidence-based decision-making regarding inventory management, resource allocation, and other aspects regarding effective supply chain system performance. An effective eELMIS can enhance availability of and access to information that is critical for decision making during PHE preparedness and response.

## Methods

We reviewed published and unpublished literature regarding the adoption and implementation of an eELMIS during the COVID-19 preparedness and response in Uganda. Documents reviewed included journal articles, reports, and national guidelines. In addition, data were abstracted from the eELMIS database. To supplement the literature review and secondary data, we held interviews and discussions with stakeholders involved in implementing the eELMIS. Furthermore, the authors used observations during the planning, preparedness, and response to the COVID-19 in Uganda (most of whom were actively involved and attended many of the meetings and participated in the epidemic response). The results are structured to capture adoption, implementation, and results of the use of the eELMIS during the COVID-19preparedness and response in Uganda.

## Results

### Uganda’s public health emergency supply chain

**Fig. 1:**
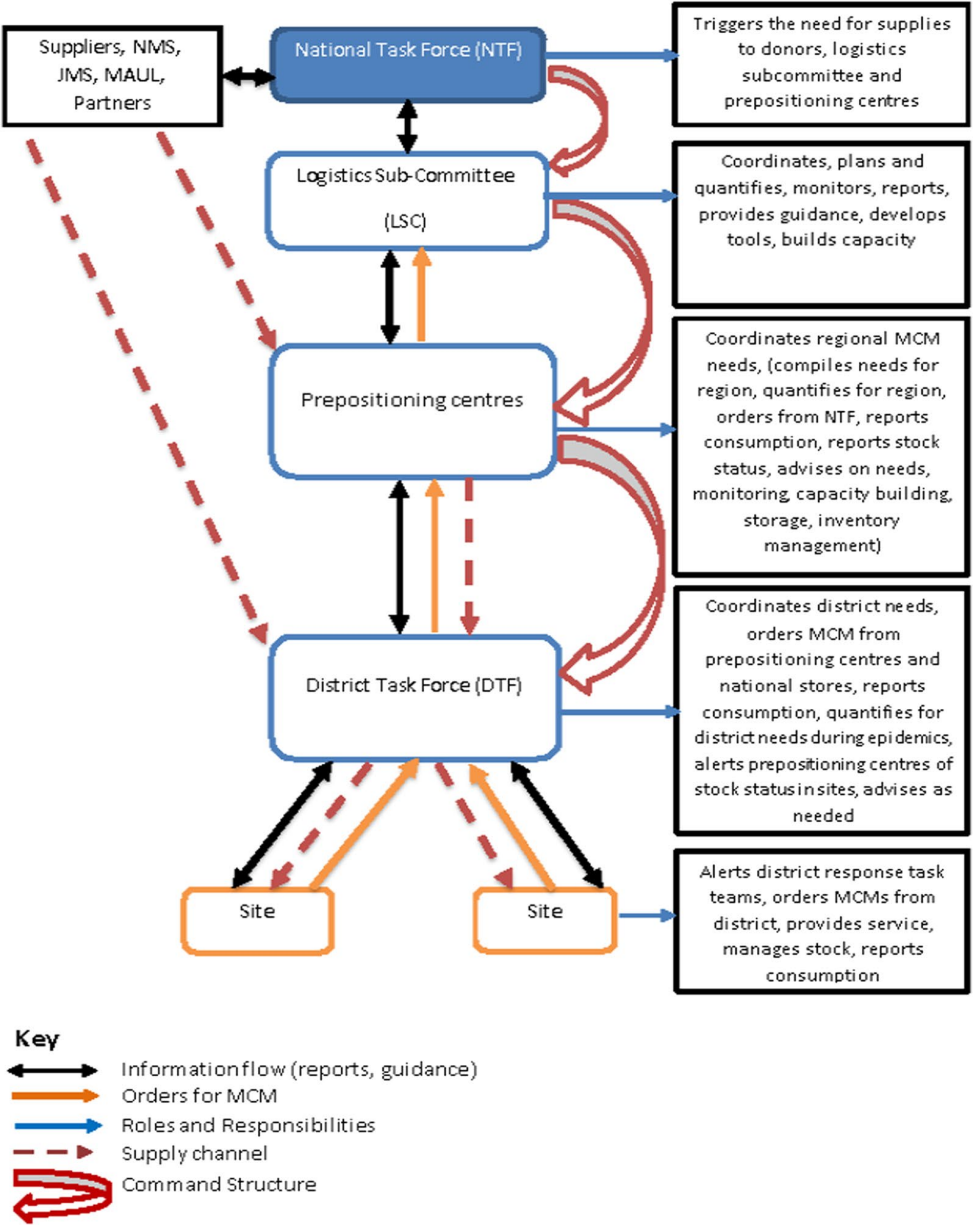
Conceptual flow of COVID-19 MCMs in the eELMIS. Source: Uganda guidelines for Managing MCMs, 2019

Uganda’s PHE supply chain system is anchored on a robust electronic information system known as the eELMIS. The eELMIS was established to support the supply chain preparedness and response to the Ebola virus disease (EVD). This system was later adopted to support preparedness and response during the COVID-19 emergency (Aceng, Ario et al. 2020). The eELMIS is a Ministry of Health (MoH) web-based information management system. The eELMIS is a standalone system specifically tailored to support the supply chain system during PHEs. The eELMIS is built with core functionalities of stock ordering, receiving issuing and reporting. These functionalities are available at four levels, i.e., the district, node, partner and national modules (Fig. 1). These modules support routine transactions of emergency supplies including COVID-19 [23]. The system facilitates tracking, ordering and distribution of supplies and commodities across the emergency supply chain system. It is designed with functionalities to track real time emergency transactions of orders, receipts, issues, and reports of emergency supplies. Through the eELMIS, health facilities order for emergency supplies on a bi-weekly basis. The expected turnaround time for delivery of the commodities from the warehouses to health facilities is four days. Furthermore, health facilities, regional prepositioning centers and partners are in position to track stock status for the different emergency supplies through the system.

### Coordination of the public health emergency supply chain system

The eELMIS supported the MoH logistics sub-committee to coordinate the supply chain system at all levels including national, regional, district, and health facilities during the COVID-19 emergency. The MoH logistics sub-committee is one of the sub-committees of the national task force charged with spearheading the coordination of the logistics functions in the country. The eELMIS supported the coordination of all partners, including the central warehouses (National Medical Stores [24] and Joint Medical Stores (JMS)), regional prepositioning centers, district stores and emergency treatment centers/units [25]. At the emergency treatment centers/units, orders for COVID-19 supplies were compiled and entered in the eELMIS by the logistics focal person and submitted to the district medicine stores. The district task force commander (District Health Officer (DHO)) is responsible for approving the orders at the district level depending on the availability of Medical Counter Measures (MCMs). If the MCMs are not available at the district stores, the DHO aggregates all the orders from the emergency treatment centers/units and submits to the regional prepositioning center or node. The node commander approves the order and issues the COVID-19 MCMs back to the district. In the absence of sufficient stock at the node, the node commander aggregates all the orders from all the districts served by the regional node (on average 20 districts) and submits to the national task force through the logistics sub-committee. The national task force approves the orders and that are allocated to a central warehouse or partner for issuing back to the node [23].

The eELMIS supported the MoH to leverage the support of different stakeholders during the COVID-19 response. Through the eELMIS different partners such as World Health Organization (WHO), World Food Programme (WFP), United Nations Children’s Fund [26], the Global Fund, International Organization for Migration (IOM), United Nations High Commission for Refugees (UNHCR) and other stakeholders made different contributions in terms of emergency stock supplies and financial support. Some of the emergency stock contributed include face masks (N95 respirators, surgical masks, and full-face shield masks), gloves (examination, surgical and heavy duty), protective goggles, fluid resistant coveralls and gowns, alcohol-based hand rub, sodium hypochlorite, oxygen concentrators, oxygen cylinders, pulse oximeters etc. The commodities were allocated by the logistics sub-committee according to the various orders/requests made by the various user units all over the country. This supported transparency and accountability and prevented duplication of efforts.

### Real time reporting and data visibility

**Fig. 2:**
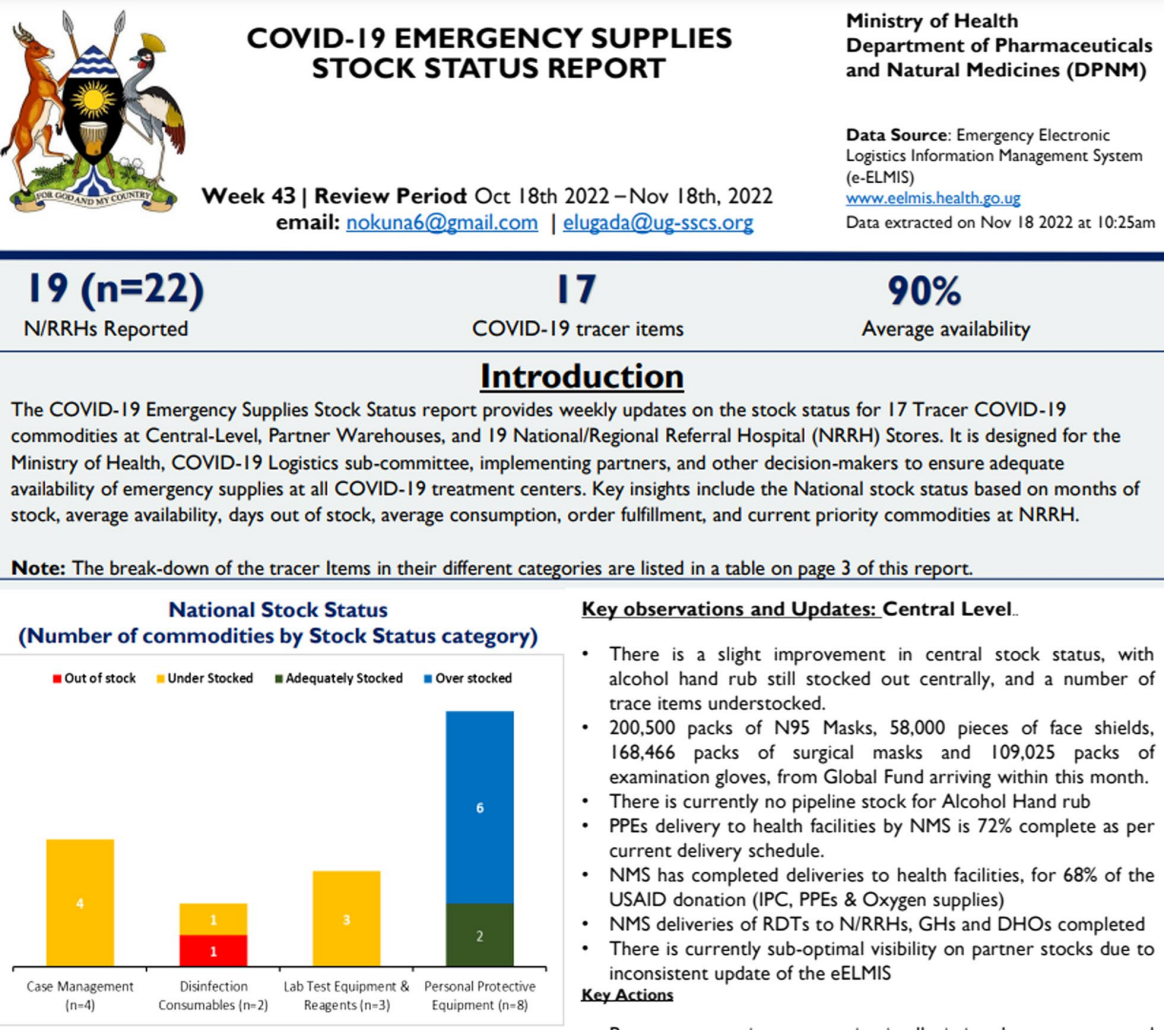
Real time stock status data at National and Regional referral hospitals (user-units) in eELMIS. Source: www.eelmis.health.go.ug

The eELMIS provided real time data on PHE commodities from user units. The data were essential for planning and management of commodities and other supplies. User units, districts, and partners are expected to update and provide weekly stock status using the eELMIS. These data are essential for informing the quantification, forecasting and pipeline monitoring of commodities at all levels of care and decision making. One of the key roles of the logistics sub-committee is to maintain an updated inventory and inform the country of the current situation of logistics and commodities during the COVID-19 response. This role was implemented using of the eELMIS. The eELMIS provides real time back-to-back tracking of logistics activities at every level of the PHE supply chain and facilitates [27]. In addition, the eELMIS facilitated the generation of daily reports to inform the logistics sub-committee on the country’s emergency stock-status or stock on hand versus the outbreak requirements and to provide a stock gap analysis to support decision making by management (Fig. 2).

Data provided by the eELMIS supported the logistics sub-committee to prepare reports that were used to update the MoH on a weekly basis. The minister in turn presented the reports to the cabinet to make decisions on resources mobilization for the COVID-19 response and larger strategies during preparedness and response.

### Capacity building in eELMIS use for COVID-19 response.

**Fig. 3:**
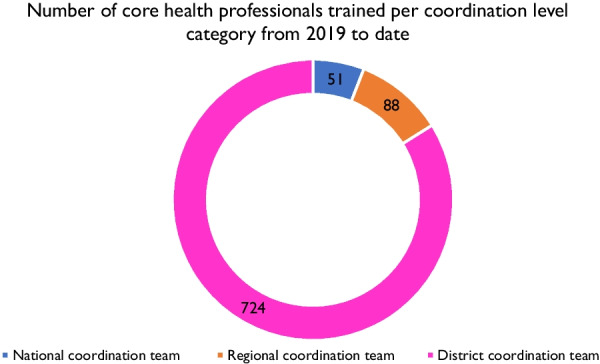
Number of health professionals trained per coordination level in PHE SCM and eELMIS use in 2019 and 2022

To strengthen the capacity of supply chain staff during the COVID-19 preparedness and response**,** about 863 staff were trained in use of the eELMIS at the national level, sub-national and health facility levels between 2019 and 2022. Staff trained at the national level consisted of members of the logistics sub-committee, donors, and partners. At the sub-national level, supply chain staff trained included those working at the regional prepositioning centers and district stores, and health facilities (emergency treatment centers). The training content included PHE supply chain management system, application, and use of the eELMIS to support the COVID-19 response. These form the core of the coordination teams at national, regional and the districts (Fig. 3). These coordination teams were trained to be Trainers of Trainers (ToTs) for Uganda in PHE supply chain management and eELMIS use, waiting to be deployed in case of any epidemic outbreak.

Under the national coordination team, partners including UNICEF, WHO, WFP, UNFPA were trained on how to use the eELMIS to inform and support their response to the COVID-19 pandemic. These partners were provided access to the eELMIS to track each entities contributions, stock-status and have it reflected in the system. This supported the logistics sub-committee in tracking partner contribution. The capacity building efforts by the logistics sub-committee are continuous and still on-going.

### Track movement of medical counter measures across the supply chain

**Fig. 4:**
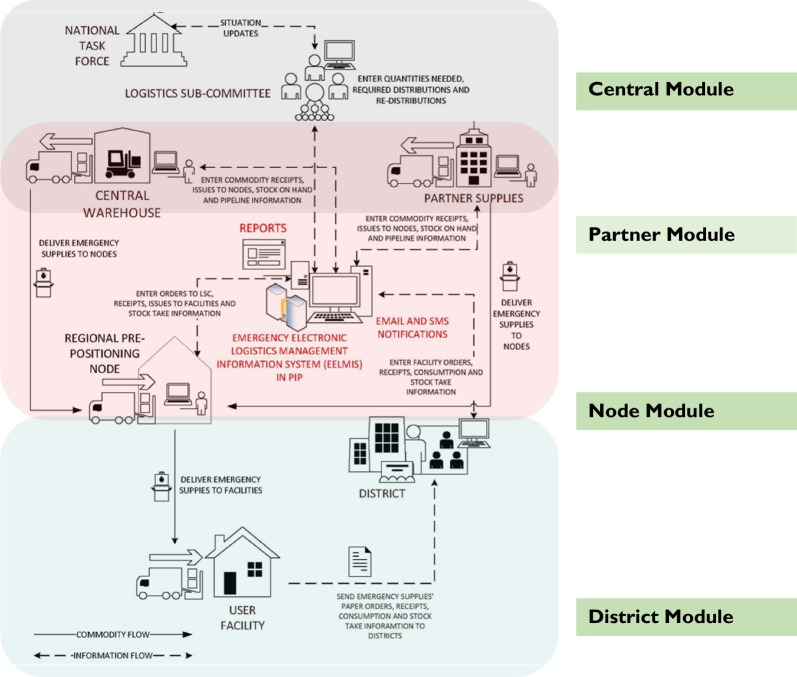
eELMIS emergency commodity flow

The eELMIS enabled the MoH to track movement of Medical Counter Measures (MCMs) from the warehouses to eight regional pre-positioning centers, and over 2000 user units (health facilities) in all 136 district medical stores. Through the eELMIS, the logistics sub-committee was able to track and coordinate COVID-19 treatment centers and prepositioning sites for MCM orders. Emergency orders for COVID-19 supplies for all 20 Regional Referral Hospitals (RRH), i.e., Mulago, Butabika, Kawempe, Kiruddu, Lira, Mubende, Mbarara, Hoima, Kabale, Moroto, Soroti, Gulu, Masaka, Jinja, Entebbe, Bombo, Mbale, Naguru, Fort portal, and Arua were processed through the eELMIS, and deliveries made to the respective hospitals. In addition to the RRHs, orders for supplies such as Personal Protective Equipment’s (PPEs) from all public hospitals in 136 districts were served through the eELMIS. Eight prepositioning sites (Mbarara, Masindi, Arua, Kasese, Mbale, Entebbe, Kotido and Lira) submitted their orders through the eELMIS to the logistics sub-committee. In addition, 89 quarantine centers and makeshift treatment centers received emergency supplies through the eELMIS. Furthermore, 75 boarders (points of entry) received emergency supplies through the eELMIS (Fig. 4).

Over 1800 bulk orders for PPEs, medicines, oxygen supplies and other non-medical COVID-19 supplies were served through the eELMIS from March 2020 to November 2022. Furthermore, 96.5% (1745) of the orders that came through the eELMIS have been served since the beginning of the response. In addition, 85.5% overall/aggregate order fulfilment rate was attained. Of all the orders that came through to the logistics sub-committee, 85.5% of the emergency supplies that were requested for were delivered.

### Quantification, forecasting, and pipeline monitoring of MCMs

The eELMIS was critical in ensuring that quality data were used for the quantification and monitoring of commodities across all levels of care. First, the Quantification Procurement and Planning Unit (QPPU) had access to consumption data and orders from all user units. This was important as it minimized shortages of emergency supplies at treatment centers as stock-outs would easily have led to higher morbidities and mortalities and further spread of the COVID-19. Second, the eELMIS supported routine planning, forecasting for future demand. In addition, it facilitated the determination of quantities of COVID-19 MCMs to be procured for both stockpiling during the preparedness and response phase, while considering the country’s supply chain, service, and resource capacities. Third, the eELMIS provided an updated catalogue list of MCMs required and available for COVID-19 response. This enabled the logistics sub-committee to support immediate deployment of COVID-19 supplies to the identified high-risk districts with point of entries, public hospitals and quarantine centers based on the quantified needs raised in the orders submitted through the eELMIS. Fourth, the eELMIS readily provided an MCM gap analysis (stock-on-hand verses outbreak requirement amount) which guided the logistics sub-committee in  resource mobilization from donors and partners (WHO, USAID, WFP, UNICEF, UNFPA, IOM etc.).

The quantification procurement and planning process was guided by an existing framework for managing MCMs for PHEs in Uganda. This framework provides details of a streamlined quantification methodology to be used during emergency response. This was efficiently referenced and followed by the QPPU which supports the logistics sub-committee. The QPPU provided daily updates to stakeholders in logistics sub-committee meetings. The logistics sub-committee meetings were held every day at the Emergency Operations Center (EOC). Issues discussed include stock-status, quantification, procurement tracking or pipeline stock information of COVID-19 emergency supplies in the country. Throughout these processes, eELMIS served as the reference tool for the QPPU.

The eELMIS supported the development of the COVID-19 emergency stock gap report that was shared and utilized to support decision making at various levels of planning and management. A report was compiled for the Commissioner pharmacy department for planning and informing budget allocations. Additionally, report was also prepared and shared with the MoH on a weekly basis by the logistics sub-committee. The minister then presented the findings to the cabinet to give the country direction on the COVID-19 preparedness and response. All this information was generated by the eELMIS as a tool for PHE supply chain during Uganda’s COVID-19 emergency response.

## Discussion

Our results demonstrate that the use of eELMIS can influence the adaptiveness of the supply chain system during PHEs. The eELMIS system supported the MoH, central medical stores, health facilities and partners adjust and respond to the increased demand for commodities brought about by the COVID-19 emergency. The use of the eELMIS contributed to the responsiveness and effectiveness of the supply chain system during the COVID-19 emergency. These findings have several implications for understanding the adaptiveness of the supply chain system in a digitally enabled extended environment and design and functionality of the eELMIS.

The eELMIS is an important information system to help the supply chain system achieve its objectives of ensuring availability of EMHS across all levels of care during PHEs. It has the potential to contribute to the overall health systems process and data exchanges thus supporting the achievement of greater integration of the supply chain system. Digital health information systems offer an opportunity to integrate entire supply chain process and functions and opening innovative ways of optimizing the logistics process [28]. In addition, it may enable real time monitoring of EMHS to react dynamically during PHEs. Further integration of the system with existing Health Information Systems offers opportunities to improve the supply chain system and enable real time information sharing that is critical during PHEs [29]. While adoption and implementation is at its early stage, the eELMIS has great potential to support not only stakeholders involved in PHE preparedness and response but the entire supply chain system. Drawing on the practical lens of implementation, these findings provides important lessons on how supply chain stakeholders can achieve process adaptability and alignment by implementing the eELMIS.

Our study advances key insights on how the eELMIS can support preparedness and response during PHEs. We show how using the eELMIS, stakeholder resources and technical capacity were amassed and deployed efficiently during the COVID-19 epidemic response. Managers, health workers and partners were able to use the eELMIS to support data-driven decision making. This is key for ensuring that emergency responders and service providers at health facilities across the country have access to critical commodities required to manage disease outbreaks. Our study shows that the use of the eELMIS supports epidemic preparedness and response. The use of the eELMIS can indeed be leveraged to build adaptive supply chain systems in the context of digitally enabled epidemic preparedness and response. As the eELMIS improves with additional capabilities with robust functionalities, to enable new architecture (such as service orientation, real-time data, and instant reporting mechanisms) we anticipate that the use of such systems will further enhance the adaptive capacity of the supply chain system particularly during PHEs.

Our findings suggest that the use of eELMIS by the different tiers of the supply chain system enables the different levels to leverage the robust capabilities and functionalities of the system. These qualities combined with a shared understanding created through the eELMIS can lead to a better understanding of the specific information needs of different supply chain stakeholders during a PHE. Similarly, by adopting and using the standard ELMIS, the eELMIS interface can build strong coordination mechanisms between different supply chain stakeholders. The use of the eELMIS enables supply chain stakeholders to know that the system is acting in the best interest of the system and that it is responsive by adopting and using the system. Thus, a standardized (yet flexible) health information systems developed and promoted to support the supply chain system is critical for reconciling both the strengths and weaknesses of existing systems. The use of eELMIS can facilitate connecting various systems without building in a digitally extended supply chain ecosystem. The eELMIS can act as a connecting tool allowing different stakeholders to bring perspectives from different parts of the system that can enhance PHE preparedness and response.

Our shows the importance of the eELMS in promoting and nurturing data use to facilitate decision making that is critical for adapting to challenges associated with preparedness and response to PHEs from a supply chain perspective. However, this necessitates investing in building capacity and increasing understanding of how the eELIMS can support different supply chain functions during PHEs. In this regard, strategy and management support are critical in facilitating the adoption, implementation of electronic logistics information systems [30]. As such the eELMIS can play a critical role capturing and disseminating knowledge and supporting its interpretation particularly when information is shared with a wide range of stakeholders with a shared agenda.

### Strength and limitations

This review had limitations as we selected articles restricted to Uganda over 5-year period which we believe is representative of the use of the electronic Logistics Management Information Systems to respond to public health emergencies in Uganda. While the literature reviewed may not be exhaustive, it is comprehensive, since it covers accessible work in the field. Notwithstanding the above limitation, we suggest future research directions on how to leverage information technology in health supply chain operations and processes to achieve competitive advantage, based on the literature and experience with other stakeholders working in this area. Future studies (using both quantitative and qualitative, cross-sectional, and longitudinal) could evaluate the effects of digital systems in different contexts (strategy, capabilities, logistics, performance) across the supply chain system.

## Conclusion

The present review showed that the eELMIS can support the supply chain system to better respond to PHEs. By supporting information management, the eELMIS ensures that the MoH and its partners have access to real time information to respond to a PHE including the increased demand for EMHS thereby ensuring responsiveness of the system. Furthermore, the findings show that it is important to build on existing health information systems that are scalable, modular, compatible and can be quickly adapted and reconfigured, thus allowing effective information flow within the supply chain system and the health system in its entirety. Furthermore, an adaptable system enables timely identification and response through its flexibility. This allows the MoH and other supply chain stakeholders to promptly meet the increased demand for commodities. While the current assessment is based on the review of reports and limited number of interviews, the findings show the importance of a cyclical loop between information generation capabilities for a simple and useful health information system that can support effective preparedness and response to PHEs.

## Data Availability

The data that support the findings of this study are available from the corresponding author upon a reasonable request.

## Abbreviations

CCHF: Crimean-Congo hemorrhagic fever
DHO: District Health Officer
eELMIS: Emergency logistics management information system
EMHS: Essential Medicines and Health Supplies
ETUs: Emergency treatment centers
EVD: Ebola, virus Disease
IOM: International Organization for Migration
LMICs: Low-and-Middle-Income-Countries
LSC: Logistics sub-committee
PHE: Public Health Emergencies
UNHCR: United Nations High Commission for Refugees
UNICEF: United Nations Children Emergency Fund
WFP: World Food Programme
WHO: World Health Organization

## References

[1] Baker RE, Mahmud AS, Miller IF, Rajeev M, Rasambainarivo F, Rice BL (2022). Infectious disease in an era of global change. Nat Rev Microbiol.

[2] Lederberg J, Hamburg MA, Smolinski MS. Microbial threats to health: emergence, detection, and response. National Academies Press; 2003. 25057653

[3] WHO. WHO Director-General’s opening remarks at the media briefing on COVID19 11-March 2020. Geneva, Switzerland: World Health Organization; 2020.

[4] World Health Organization Coronovirus (COVID-19) Dashboard [Internet]. World Health Organiztaion. 2022. Available from: https://covid19.who.int/.

[5] Piot P, Soka MJ, Spencer J (2019). Emergent threats: lessons learnt from Ebola. Int Health.

[6] Lucas DN, Bamber J (2021). Pandemics and maternal health: the indirect effects of COVID-19. Anaesthesia.

[7] WHO. International health regulations (2005): World Health Organization; 2008.

[8] Li Z, Jones C, Ejigu GS, George N, Geller AL, Chang GC (2021). Countries with delayed COVID-19 introduction–characteristics, drivers, gaps, and opportunities. Glob Health.

[9] Velásquez G, Syam N. A new WHO international treaty on pandemic preparedness and response: can it address the needs of the global south? South Centre Policy Brief. 2021;93.

[10] Nkengasong JN, Mankoula W (2020). Looming threat of COVID-19 infection in Africa: act collectively, and fast. The Lancet.

[11] Gilbert M, Pullano G, Pinotti F, Valdano E, Poletto C, Boelle P-Y, et al. Preparedness and vulnerability of African countries against introductions of 2019-nCoV. 2020. 10.1016/S0140-6736(20)30411-6 PMC7159277 32087820

[12] Hassan Z, Hashim MJ, Khan G. Population risk factors for COVID-19 deaths in Nigeria at sub-national level. Pan Afr Med J. 2020;35(Suppl 2). 10.11604/pamj.supp.2020.35.131.25258 PMC7608767 33193946

[13] Paintsil E (2020). COVID-19 threatens health systems in sub-Saharan Africa: the eye of the crocodile. J Clin Invest.

[14] Michaud CM. Global Burden of Infectious Diseases. Encyclopedia of Microbiology. 2009:444–54.

[15] Hasan MM, dos Santos Costa AC, Xenophontos E, Mohanan P, Bassey EE, Ahmad S (2021). Lassa fever and COVID-19 in Africa: a double crisis on the fragile health system. J Med Virol.

[16] Auerbach J, Miller BF. COVID-19 exposes the cracks in our already fragile mental health system. Am Public Health Assoc. 2020; 969–70. 10.2105/AJPH.2020.305699 PMC7287559 32271609

[17] Tessema GA, Kinfu Y, Dachew BA, Tesema AG, Assefa Y, Alene KA (2021). The COVID-19 pandemic and healthcare systems in Africa: a scoping review of preparedness, impact and response. BMJ Glob Health.

[18] WHO. Toolkit for Assessing Health-system Capacity for Crisis Management: Strengthening Health-system Emergency Preparedness: Parth 2. Assessment Form: World Health Organization, Regional Office for Europe; 2012.

[19] Gereffi G (2020). What does the COVID-19 pandemic teach us about global value chains? The case of medical supplies. J Int Business Policy.

[20] Akinyemi OO, Popoola OA, Fowotade A, Adekanmbi O, Cadmus EO, Adebayo A (2021). Qualitative exploration of health system response to COVID-19 pandemic applying the WHO health systems framework: case study of a Nigerian state. Scientific African.

[21] Dzinamarira T, Dzobo M, Chitungo I (2020). COVID-19: a perspective on Africa’s capacity and response. J Med Virol.

[22] Naudé W, Vinuesa R (2021). Data deprivations, data gaps and digital divides: lessons from the COVID-19 pandemic. Big Data Soc.

[23] Wasswa JH, Oteba MO, Katumba A. Uganda’s Public Health Emergency Supply Chain System in the Awake of COVID-19 Emergency Response: Method and Performance.

[24] NMS. National Medical Stores Strategic Plan 2020/21 to 2024/25. Kampala, Uganda: National Medical Stores; 2020.

[25] MoH. National Medical Countermeasures Supply-Chain Plan. Kampala, Uganda: Ministry of Health; 2020.

[26] UNICEF. The National Budget Framework FY 2019/20 Budget Brief No 2019/3 (Source: NBFP FY 2019/20–2023/24). Kampala, Uganda: UNICEF; 2019.

[27] John H, Henry O, Jude O, Ahmed K. The role of the eELMIS as a tool of public health emergency supply chain management during Uganda’s COVID-19 Emergency Response. Int J Sci Res. 2020;9(7).

[28] Bastani P, Dehghan Z, Kashfi SM, Dorosti H, Mohammadpour M, Mehralian G (2021). Strategies to improve pharmaceutical supply chain resilience under politico-economic sanctions: the case of Iran. J Pharm Policy Practice.

[29] Nguyen NE. A case study investigating integration and interoperability of Health Information Systems in sub-Saharan Africa 2019.

[30] Darabi S, Iranpoor M, Amindoust A. The critical success factors for implementing eletronic lohistics in the Isfahan health city. J Healthc Manag. 2016;7(3).

